# Climate-control ChatGPT for EFL writing and AI literacy

**DOI:** 10.3389/frai.2026.1869343

**Published:** 2026-07-22

**Authors:** Hesham Aldamen, Salwa Mohammad Alawneh, Mohamad Almashour, Mutasim Al-Deaibes, Rami Alsharefeen

**Affiliations:** 1 Department of English Language and Literature, The University of Jordan, Amman, Jordan; 2 Department of Architectural Engineering, Yarmouk University, Irbid, Jordan; 3 Department of English, American University of Sharjah, Sharjah, United Arab Emirates; 4 Faculty Affairs, Rabdan Academy, Abu Dhabi, United Arab Emirates

**Keywords:** AI literacy, anthropomorphism, ChatGPT, EFL writing, HARM-CAL, harm-sensitive reasoning, natural language processing, second language argumentation

## Abstract

ChatGPT is increasingly used in EFL writing, yet applied linguistics lacks robust methods for measuring whether human-AI dialogue improves learners’ AI literacy, harm-sensitive reasoning, and independent argumentative writing. This mixed-methods quasi-experimental study examines whether climate-control ChatGPT, a prompted interactional design that requires learners to justify, qualify, counterexample, and re-author their own claims, produces stronger gains than standard ChatGPT use and no-ChatGPT instruction. The study involved 120 undergraduate EFL learners in six intact second-year academic writing sections at a public university in Jordan. Participants were assigned to no-ChatGPT control, standard ChatGPT, or climate-control ChatGPT conditions. Data included no-AI diagnostic, pretest, posttest, and delayed transfer essays; ChatGPT dialogue logs; first and final drafts; learner reflections; uptake traces; stimulated-recall interviews; and 9,113 HARM-CAL annotated language units. HARM-CAL, Harm-sensitive Reasoning and Anthropomorphism Calibration in Language, classified learner language for naïve relativism, moral imposition, harm-sensitive reasoning, AI anthropomorphism, calibrated AI literacy, and reflective uncertainty. Results showed that the climate-control condition produced the largest gains in harm-sensitive AI literacy and argumentative writing quality, with advantages maintained on delayed no-AI transfer. It also reduced direct uptake, increased self-repair and authorial control, and strengthened calibrated distinctions between AI output competence and consciousness. The study contributes a classroom-tested intervention and a transparent computational framework that combines reproducible annotation procedures, a fully specified TF-IDF baseline, and archived transformer-family classifier outputs for analyzing responsible AI literacy in second-language argumentation.

## 1 Introduction

Generative artificial intelligence has entered second-language writing classrooms faster than applied linguistics has developed methods for measuring what such interaction changes in learner reasoning. ChatGPT can help learners generate ideas, reformulate sentences, organize drafts, and receive rapid language support, and recent research has reported promising uses in L2 writing, feedback, learner motivation, and classroom assessment (Barrot, 2023; Kohnke et al., 2023; Li et al., 2024; Lu et al., 2024; Song and Song, 2023; Su et al., 2023; Yan, 2023; Yang and Li, 2024; Aldamenet al., 2026a; Aldamen et al., 2025). These contributions are important, but they leave a more difficult question unresolved. If learners produce stronger essays after using ChatGPT, does this improvement reflect development in their own English reasoning, or does it partly reflect access to fluent external language?

This question matters because writing quality and reasoning quality are not identical. A learner may produce a polished paragraph that is weakly justified, conceptually vague, or overly dependent on AI-generated phrasing. Conversely, a less fluent draft may reveal genuine development in claim formation, warranting, qualification, and counterargument. This distinction is especially important in EFL contexts, where learners may reasonably value ChatGPT for linguistic fluency while also becoming vulnerable to direct uptake, reduced authorial control, or unexamined acceptance of AI-generated claims (Barrot, 2023; Perkins, 2023; Praphan and Praphan, 2023; Yan, 2023, Aldamen et al., 2026b). The central issue is therefore not whether ChatGPT can improve the surface form of learner writing. It is whether human-ChatGPT dialogue can support transferable growth in how learners reason in English.

The present study addresses this issue through the domain of harm-sensitive artificial-intelligence literacy. This construct refers to learners’ ability to reason in English about AI-related ethical issues while distinguishing problem-solving intelligence from consciousness, suffering, moral standing, and entitlement. It is not treated here as a general attitude toward AI or as familiarity with digital tools. It is a language-mediated reasoning capacity visible in claims, warrants, hedges, contrasts, counterexamples, and explanations of harm. This focus is necessary because fluent language models can produce persuasive moral language without understanding, consciousness, or suffering in the human sense (Bender and Koller, 2020; Bender et al., 2021; Dehaene et al., 2017). Learners who infer feeling, intention, empathy, or moral authority from fluent output may anthropomorphize AI systems in ways that weaken ethical reasoning. Anthropomorphism is therefore not only a philosophical concern. It is also a linguistic and pedagogical risk, since it appears in the verbs, predicates, and explanations learners use when writing about AI (Epley et al., 2007).

The study introduces climate-control ChatGPT as a response to this problem. A standard chatbot often provides polished explanations, examples, and arguments. Such support can be useful, but it may also make it easier for learners to borrow claims before they have generated their own reasoning. A climate-control tutor is designed differently. It changes the conditions of interaction by asking learners to state a claim first, identify assumptions, explain who may be harmed, distinguish intelligence from consciousness, consider counterexamples, qualify conclusions, and produce the final wording themselves. It does not provide model essays, full paragraph replacements, moral verdicts, or ready-made conclusions. In this design, ChatGPT is not treated as an answer generator but as a constrained reasoning environment.

The study also introduces HARM-CAL, Harm-sensitive Reasoning and Anthropomorphism Calibration in Language, as a computational framework for measuring these changes. HARM-CAL classifies learner-produced language units into six multi-label reasoning states: naïve relativism, moral imposition, harm-sensitive reasoning, AI anthropomorphism, calibrated AI literacy, and reflective uncertainty. The framework is designed to make applied-linguistic interpretation computationally tractable without reducing reasoning to keyword counts. It links human annotation, reliability analysis, supervised classification, uptake coding, and qualitative explanation. This approach responds to the need for transparent computational methods in research on language learning and artificial intelligence, especially methods that make annotation procedures, reliability evidence, baseline models, and reproducibility boundaries explicit. (Artstein and Poesio, 2008; Gebru et al., 2021; Mitchell et al., 2019; Papi et al., 2024; Storks et al., 2023). Climate-control ChatGPT and HARM-CAL place the inquiry at the intersection of AI-mediated language learning, computational analysis of learner language, and second-language argumentation: the classroom interaction supplies the pedagogical design, while the annotated units and classifier make the language evidence testable.

The study reports a quantitative-dominant explanatory mixed-methods quasi-experimental investigation with 120 undergraduate EFL learners in a second-year academic writing course at a public university in Jordan. Six intact sections were assigned to no-ChatGPT control, standard ChatGPT, or climate-control ChatGPT conditions. The dataset includes no-AI diagnostic, pretest, posttest, and delayed transfer essays; ChatGPT dialogue logs; first and final intervention drafts; learner reflections; HARM-CAL annotated units; uptake traces; model predictions; and stimulated-recall interviews with a focal subsample. This design allows the study to examine not only whether learners improved, but also how improvement appeared in dialogue, revision, uptake, computationally classified reasoning states, and later independent writing.

The study was guided by the following research questions:

1. Does climate-control ChatGPT produce stronger gains than standard ChatGPT and no-ChatGPT instruction in EFL learners’ harm-sensitive artificial-intelligence literacy and English argumentative quality?
2. Can HARM-CAL reliably classify reasoning states in Jordanian EFL learner essays and student-ChatGPT dialogue?
3. Which linguistic and interactional features distinguish productive climate-control dialogue from standard ChatGPT dialogue?
4. How do learners explain moments in which ChatGPT helps them revise moral disagreement, harm, suffering, and AI consciousness in English?

## 2 Related work and conceptual framework for ChatGPT and AI literacy

### 2.1 ChatGPT, EFL writing, and measurable learning

Research on generative AI in second-language writing has moved quickly from curiosity about access to more specific questions about pedagogy, feedback, authorship, and assessment. Broader work on artificial intelligence in higher education similarly frames large language models as both pedagogical opportunities and sources of risk (Kasneci et al., 2023; Ouyang et al., 2022). ChatGPT has been described as useful for idea generation, language reformulation, feedback, revision, and learner confidence, especially in contexts where students need frequent support but teachers cannot provide individualized feedback at every stage of composing (Alnemrat et al., 2025; Barrot, 2023; Kohnke et al., 2023; Song and Song, 2023; Yan, 2023; Yang and Li, 2024). Studies of AI-assisted writing have also shown that learners may use ChatGPT to clarify task demands, develop arguments, improve cohesion, and notice language problems that they might otherwise overlook (Li et al., 2024; Lu et al., 2024; Su et al., 2023).

At the same time, this body of work has raised concerns about dependency, academic integrity, authorship, inaccurate feedback, and the risk that polished AI language may conceal weak reasoning (Barrot, 2023; Perkins, 2023; Praphan and Praphan, 2023; Yan, 2023). These concerns are especially important in EFL writing because learners may accept fluent AI-generated language as evidence that an argument is conceptually strong. A sentence can sound academic while still lacking a warrant, misrepresenting a counterargument, or treating a vague claim as if it were justified. For this reason, the central problem is not simply whether ChatGPT improves the surface quality of writing. The more difficult question is whether human-ChatGPT dialogue changes the structure of learners’ reasoning in English.

This study builds on prior L2 writing research but shifts the unit of interest. Much previous work has focused on final texts, feedback quality, learner perceptions, or writing scores (Barrot, 2023; Song and Song, 2023; Su et al., 2023; Yan, 2023). These outcomes remain important, but they do not fully capture the process by which a learner moves from a first claim to a revised argument. Writing development also involves stance, lexical precision, cohesion, counterargument, qualification, and the ability to support claims with reasons (Aull and Lancaster, 2014; Kyle and Crossley, 2015; McNamara et al., 2010; Polio, 2017). In AI-mediated writing, those abilities need to be studied not only in final essays but also in learner turns, AI responses, draft changes, uptake decisions, and later no-AI transfer.

Digital writing-support research provides a useful foundation for this shift. Earlier systems showed that automated writing tools can support strategy instruction and textual improvement when they are designed to guide revision rather than merely correct local errors (McNamara et al., 2013; Strobl et al., 2019). ChatGPT differs from earlier tools because it can generate extended, context-sensitive prose, but that affordance is pedagogically double-edged. It can make support more flexible, yet it can also make learner dependence less visible. The present study therefore treats ChatGPT not as a neutral writing aid but as an interactional environment whose design may either preserve or weaken learner agency.

### 2.2 From AI writing support to language-mediated reasoning

The study is grounded in the view that argumentation in a second language is not only a matter of linguistic accuracy. It is also a form of language-mediated reasoning. Learners must select claims, connect those claims to evidence, anticipate objections, qualify conclusions, and manage uncertainty. These reasoning practices are visible in linguistic choices such as causal connectives, contrast markers, hedges, stance expressions, and explanatory clauses (Aull and Lancaster, 2014; McNamara et al., 2010). When ChatGPT enters this process, it can influence both language and reasoning. It may supply vocabulary, transitions, examples, and counterarguments, but it may also shape what the learner treats as a sufficient reason.

This distinction matters because studies of ChatGPT in language learning have often reported improvements in writing performance, motivation, or perceived usefulness without fully separating learner-generated reasoning from AI-supported textual production (Kohnke et al., 2023; Song and Song, 2023; Yang and Li, 2024). If learners use AI-generated claims directly, an improved essay may partly reflect access to external argumentation rather than development in the learner’s own reasoning. Conversely, if learners revise, reject, or re-author AI suggestions, ChatGPT may become a productive scaffold for reasoning. The same tool can therefore produce different learning consequences depending on the structure of interaction.

This study addresses that problem by examining no-AI pretest, posttest, and delayed transfer essays alongside dialogue logs, drafts, uptake traces, reflections, and stimulated-recall evidence. The delayed no-AI task is especially important because it tests whether learners can reproduce improved reasoning without immediate AI support. The study therefore distinguishes between AI-assisted performance and transferable development. This distinction is consistent with broader concerns in second-language writing research about how to interpret textual improvement when learners receive different forms of external support (Polio, 2017).

### 2.3 Harm-sensitive AI literacy

The conceptual focus of the study is harm-sensitive artificial-intelligence literacy. In this study, the construct refers to the learner’s ability to reason in English about AI-related ethical issues while distinguishing problem-solving intelligence from consciousness, suffering, moral standing, and political or legal entitlement. It is not treated as a general attitude toward AI, a measure of technological familiarity, or a test of agreement with one philosophical position. It is a language-mediated reasoning capacity that appears in argument structure, lexical choices, explanations of harm, hedging, counterexamples, and resistance to unsupported anthropomorphic claims. A learner can advance a speculative or nonstandard position about future AI moral status and still demonstrate strong reasoning if the claim is explicitly warranted, qualified, and evidence-sensitive.

The harm-sensitive dimension is important because ethical reasoning about AI often becomes vague when learners rely only on opinion, cultural difference, or authority. Moral psychology has shown that moral judgment can involve emotional, intuitive, and deliberative processes (Greene et al., 2001; Haidt, 2001). In a writing classroom, however, students must turn judgment into public reasoning. They need to explain who may be harmed, how harm occurs, what evidence supports the claim, and what limits apply. A harm-sensitive argument does not require moral certainty. It requires the learner to move beyond “people disagree” or “this is wrong” toward an explicit account of suffering, vulnerability, consequences, and justification.

The AI-literacy dimension adds another layer. Large language models can generate fluent and persuasive text without understanding language in the human sense (Bender and Koller, 2020; Bender et al., 2021). This distinction is central to the study because learners may infer consciousness, intention, empathy, or moral insight from fluent output. Harm-sensitive AI literacy therefore requires students to distinguish what an AI system can do from what that performance proves about inner experience or moral status. In this framework, calibrated AI literacy does not require learners to repeat a fixed ethical verdict. It requires them to make the basis of their claim visible: what evidence is available, what remains uncertain, who or what can be harmed, and why fluency alone is insufficient evidence of feeling, suffering, or moral standing.

### 2.4 Anthropomorphism as a linguistic and pedagogical risk

Anthropomorphism is a major conceptual risk in human-AI interaction. People often attribute humanlike qualities to nonhuman agents, especially when those agents display apparent responsiveness, social behavior, or communicative fluency (Epley et al., 2007). ChatGPT intensifies this risk because it produces language that resembles explanation, empathy, advice, and reasoning. In EFL writing, the problem is not only philosophical. It is also linguistic. Learners may use verbs such as understands, feels, believes, wants, or suffers in ways that imply mental states without evidence.

The distinction between intelligence and consciousness is central here. Cognitive and neuroscientific accounts of consciousness caution against equating problem-solving ability or information processing with subjective experience (Dehaene et al., 2017). This distinction is especially relevant when learners discuss whether AI can be harmed, respected, blamed, or granted moral standing. A system may generate a convincing response about suffering without itself suffering. It may produce moral language without being a moral patient. If learners do not control these distinctions linguistically, their arguments may become conceptually unstable.

This study therefore treats anthropomorphism as both a reasoning-state marker and a language problem. AI anthropomorphism is not coded simply because a learner uses a metaphor, a convenient shorthand, or a carefully argued philosophical position about nonhuman agency. It becomes analytically important when the learner attributes feeling, suffering, intention, moral insight, or entitlement to AI without warrant. By contrast, calibrated AI literacy appears when the learner distinguishes output competence from consciousness or suffering, or when a speculative claim is explicitly marked as conditional, evidentially limited, and open to future revision. These categories allow the study to examine whether ChatGPT-supported writing encourages or reduces unsupported anthropomorphic drift without treating culturally or philosophically diverse reasoning as deficient by default.

### 2.5 Climate-control ChatGPT

Climate-control ChatGPT is the intervention construct developed for this study. It refers to a prompted interactional design in which ChatGPT is instructed to create conditions for learner reasoning rather than provide finished answers. A climate-control tutor asks the learner to state a claim before receiving suggestions, requests warrants and counterexamples, prompts distinctions between intelligence and consciousness, asks who can suffer and how harm occurs, discourages unsupported anthropomorphism, and requires the learner to write the final wording. It does not supply model essays, full paragraphs, moral verdicts, or ready-made conclusions.

The term climate-control is used because the intervention does not simply add feedback to writing. It changes the conditions under which writing and reasoning occur. Standard ChatGPT can quickly provide polished arguments. That speed may be useful, but it can also reduce the learner’s need to generate claims, test limits, or repair weak reasoning. Climate-control prompting deliberately slows this process. It is designed to preserve productive difficulty by requiring the learner to do the cognitive and linguistic work that matters for development.

This construct responds to a gap in the AI-assisted writing literature. Several studies have shown that ChatGPT can support writing or feedback, but fewer have examined how prompt architecture may regulate learner agency and reasoning quality (Barrot, 2023; Su et al., 2023; Yan, 2023; Yang and Li, 2024). The present study proposes that the pedagogical effect of ChatGPT depends not only on the model’s capabilities but also on the interactional rules imposed on it. A model that gives answers and a model that asks disciplined questions may produce different learning outcomes even when the underlying technology is similar.

### 2.6 The HARM-CAL classification framework

HARM-CAL, or Harm-sensitive Reasoning and Anthropomorphism Calibration in Language, is the computational framework used to operationalize the study’s conceptual claims. It classifies learner-produced language units into six multi-label reasoning states: naïve relativism, moral imposition, harm-sensitive reasoning, AI anthropomorphism, calibrated AI literacy, and reflective uncertainty. These labels are multi-label because learner language can carry more than one reasoning function at the same time. A student may, for example, express reflective uncertainty while also making a harm-sensitive distinction.

The framework is designed to make applied-linguistic interpretation computationally tractable without reducing it to simple keyword counting. HARM-CAL units are clauses, sentences, or short turns that express claims, warrants, contrasts, uncertainty markers, anthropomorphic attributions, or AI-literacy distinctions. The labels therefore remain anchored in language. They do not claim to identify private beliefs or moral character. They identify textual evidence of reasoning states that can be annotated, checked for reliability, and modeled computationally.

This approach draws on standards in computational linguistics and NLP that emphasize reliable annotation, transparent evaluation, and careful interpretation of model performance. Inter-coder agreement is essential when human judgments form the basis of computational classification (Artstein and Poesio, 2008; Cohen, 1960). Transparent NLP also requires clear documentation of datasets, models, baselines, software environments, reproducibility boundaries, and known limitations (Gebru et al., 2021; Mitchell et al., 2019; Papi et al., 2024; Storks et al., 2023). The present study therefore evaluates HARM-CAL through human reliability, a supervised transformer-family classifier, a fully specified TF-IDF logistic-regression baseline, participant-level cross-validation, and label-specific error analysis. Sentence-level representation methods are appropriate for this kind of short-unit classification because they are designed to capture semantic relations in compact textual segments (Reimers and Gurevych, 2019). In the present study, reproducibility is strongest for the annotation framework, segmentation rules, participant-level cross-validation structure, and TF-IDF baseline; transformer-family results are reported as archived benchmark outputs rather than as fully checkpoint-reproducible results.

The framework also serves the mixed-methods logic of the study. Quantitative scores show whether learners improve. HARM-CAL distributions show how reasoning states shift across time and condition. Uptake coding shows whether AI language moves into learner drafts directly or through revision and re-authorship. Reflections and stimulated recall explain how learners understood those moments. This integration follows the view that mixed-methods research is strongest when different forms of evidence are connected through design, analysis, and interpretation rather than merely reported side by side (Fetters et al., 2013; Guetterman et al., 2015).

### 2.7 Conceptual synthesis

The conceptual framework links four claims. First, ChatGPT can support L2 writing, but its educational value depends on how it structures learner agency, uptake, and reasoning. Second, harm-sensitive AI literacy is a language-mediated reasoning capacity that requires learners to distinguish harm, suffering, consciousness, moral standing, and output competence. Third, anthropomorphism is not only a philosophical concern; it is a linguistic risk visible in the predicates and explanations learners use when writing about AI. Fourth, HARM-CAL offers a way to measure these reasoning states across essays, dialogue turns, drafts, and transfer tasks.

On this basis, the study treats climate-control ChatGPT as a designed reasoning environment and HARM-CAL as the analytic bridge between classroom interaction and computational evidence. The central expectation is that learners who interact with a tutor constrained to ask, challenge, and require re-authorship will show stronger delayed no-AI gains than learners who use ChatGPT as a general writing-support tool. The expected evidence is not only higher rubric scores, but also increased harm-sensitive reasoning, greater calibrated AI literacy, lower unsupported anthropomorphism, reduced direct uptake, and richer learner explanations of revision and authorial control. In this sense, learner-ChatGPT dialogue is treated not only as a classroom event but also as a language-computation problem: the same learner claims, hedges, warrants, and revisions must be interpretable to teachers and operational enough to support annotation, reliability testing, and supervised classification.

## 3 Materials and methods

### 3.1 Research design

The study used a quantitative-dominant explanatory mixed-methods quasi-experimental design with a computational language-processing core. The design was quasi-experimental because six intact second-year academic writing sections were assigned to one of three instructional conditions: no-ChatGPT control, standard ChatGPT, or climate-control ChatGPT. It was mixed-methods because the study estimated measurable change in learner writing and AI-literacy outcomes while also examining how such change appeared to develop through dialogue, revision, reflection, and stimulated recall. It was computational because the study developed and evaluated HARM-CAL, a multi-label framework for classifying harm-sensitive reasoning and anthropomorphism calibration in second-language English argumentation.

The design was selected because the study did not treat ChatGPT use as a simple exposure variable or evaluate only final essay quality. The main object of analysis was the learner’s language-mediated reasoning across a classroom ecology that included no-AI writing, AI-supported dialogue, draft revision, learner reflection, human annotation, and supervised NLP classification. The explanatory mixed-methods structure allowed quantitative and computational patterns to be interpreted through qualitative evidence from learner reflections and stimulated-recall interviews (Fetters et al., 2013; Guetterman et al., 2015). HARM-CAL was therefore used as a transparent analytic bridge between applied-linguistic interpretation and computational modeling, with reproducible annotation, reliability, cross-validation, and baseline-model components, not as a substitute for human judgment.

### 3.2 Context and participants

The study was conducted in a required second-year undergraduate academic writing course in a Department of English at a public university in Jordan. This course level was selected because second-year EFL writers are generally able to produce extended argumentative texts while still developing control of warrants, counterarguments, hedging, qualification, and conceptual precision. First-year students were likely to introduce excessive variation in basic essay organization, whereas third- and fourth-year students were more likely to differ substantially in prior disciplinary writing experience and independent AI-use practices.

The analytic sample consisted of 120 undergraduate EFL learners distributed across six intact sections, with 20 students in each section. Two sections were assigned to each instructional condition, producing 40 participants in the no-ChatGPT control condition, 40 in the standard ChatGPT condition, and 40 in the climate-control ChatGPT condition. All participants were anonymized before analysis using codes from P01 to P120. The analytic files used these codes for essays, dialogue logs, reflections, interview excerpts, scoring sheets, annotation records, uptake traces, and model predictions.

A qualitative focal subsample of 24 students was selected for stimulated recall, with eight participants from each condition. Selection was purposive rather than random. After preliminary pretest scoring, focal learners were chosen to represent high, middle, and low baseline writing performance as well as different early reasoning profiles. This sampling strategy supported explanatory range across cases while keeping the qualitative strand closely linked to the study’s quantitative and computational evidence.

### 3.3 Proficiency determination and inclusion criteria

Participants were treated as upper-intermediate EFL learners, operationalized as CEFR B1 + to B2. Proficiency was determined through two Week 1 measures. First, all consenting students completed the Oxford Online Placement Test under supervised conditions. The continuous placement score was retained as the main proficiency covariate. Second, students completed a 35-min no-AI diagnostic argumentative writing task, which was scored with the study writing rubric. The diagnostic score was used to confirm writing-specific baseline comparability and to support sensitivity analysis.

Students whose placement and diagnostic evidence placed them clearly below B1 + or above B2 were excluded from the analytic dataset, although they continued normal course participation. This restriction reduced proficiency-related noise in the estimation of writing development, AI-literacy gains, and HARM-CAL reasoning-state distributions. It also helped ensure that observed differences were not simply artifacts of highly uneven English proficiency.

### 3.4 Condition assignment and contamination control

The intact section was the unit of assignment. The six sections were assigned to the three conditions in matched pairs after scheduling constraints were known. The same instructor, syllabus, instructional sequence, prompts, rubrics, time limits, and classroom materials were used across the sections. The no-ChatGPT control group completed study tasks in monitored computer labs with access restricted to the writing platform and permitted dictionaries. The two ChatGPT groups used study-created ChatGPT accounts in supervised lab sessions. Memory was disabled, the displayed model name and version were recorded at the time of data collection, and all prompts and outputs were archived.

Several procedures were used to reduce contamination. Study tasks were completed during scheduled sessions, and students were instructed not to share prompts, outputs, or drafts across sections. No-AI writing tasks were completed with internet access disabled. Because ordinary exposure to generative AI cannot be fully eliminated in contemporary university settings, the study focused on supervised task performance, archived interaction records, and condition-specific instructional procedures rather than assuming total insulation from outside AI use. This choice reflects the practical classroom conditions described in recent work on ChatGPT and L2 writing (Barrot, 2023; Kohnke et al., 2023; Yan, 2023).

### 3.5 Intervention conditions

The no-ChatGPT control condition received ordinary academic writing instruction on argument structure, claim-warrant-evidence relations, counterargument, hedging, paragraph cohesion, and language control. Students in this condition did not use ChatGPT for study tasks. This condition established the baseline effect of regular writing instruction.

The standard ChatGPT condition received a short orientation on appropriate procedural use of ChatGPT. Students were allowed to ask ChatGPT for help understanding prompts, generating ideas, improving reasoning, revising wording, and checking clarity. They were prohibited from asking for a complete essay or full paragraph replacement. They were required to submit their own final wording and to retain a complete record of prompts and outputs. This condition represented plausible classroom use of ChatGPT as a general writing-support tool.

The climate-control ChatGPT condition used a standardized system prompt. ChatGPT was instructed to act as a reasoning tutor rather than an answer provider. The tutor had to ask one question at a time, require the learner to articulate a claim before receiving suggestions, request warrants and counterexamples, ask about limits and evidence, distinguish AI intelligence from consciousness, suffering, and moral standing when relevant, discourage unsupported anthropomorphic claims, and ask who can suffer, how harm occurs, and why harm matters. It was not permitted to write full answers, model essays, full paragraph replacements, or moral verdicts. It also had to require the learner to produce the final wording. The climate-control condition was therefore designed to preserve learner agency, justification, and authorial control while still allowing AI-supported dialogue.

### 3.6 Writing tasks, dialogue cycles, and data sources

The study included four no-AI writing tasks and three intervention dialogue-writing cycles. The no-AI tasks consisted of a Week 1 diagnostic essay, a pretest, an immediate posttest, and a delayed transfer task four weeks after the intervention. These tasks were completed in supervised settings without AI access. The prompts addressed responsible AI use in academic writing, moral disagreement and harm, AI intelligence versus consciousness, and university teaching of responsible AI use while protecting learner agency and authorial control.

The three intervention dialogue-writing cycles were completed during normal course sessions. They focused on conceptually linked prompts concerning moral disagreement and harm, AI intelligence versus consciousness, and educational environments that create or suppress learner agency when students use language technologies. Direct religious framing was excluded from all prompts. The prompts invited reasoning about suffering, evidence, limits, consciousness, moral standing, and agency without asking students to disclose private religious, political, medical, or personal moral beliefs.

Table 1 summarizes the main data sources, corpus size, units of analysis, and analytic functions.

**Table 1:** Main data sources and analytic functions.

Data source	Rows	Unit of analysis	Analytic function
Participants	120	Participant	Sample structure, condition assignment, proficiency covariates
Sections	6	Intact section	Blocking structure for quasi-experimental assignment
Prompts	7	Study prompt	Linkage across writing, dialogue, draft, and reflection tasks
No-AI writing scores	480	Participant-task score	Primary literacy and argumentative writing outcomes
No-AI essays	480	Participant-task essay	Independent writing and reasoning evidence
Intervention drafts	720	Participant-cycle-draft stage	Revision, authorial control, and draft development
ChatGPT logs	3,108	Dialogue turn	Learner-AI interaction, prompting, and reasoning process
ChatGPT session summaries	240	Participant-cycle session	Session-level dialogue features
Learner reflections	360	Participant-cycle reflection	Learner explanations of revision, ownership, and reasoning change
HARM-CAL units	9,113	Argument-relevant language unit	Human annotation and NLP classification
Double-coded HARM-CAL units	2,278	Argument-relevant language unit	Inter-rater reliability estimation
Uptake coding	967	Uptake trace	Movement of ChatGPT wording or claims into learner drafts
Stimulated-recall interviews	120	Interview excerpt	Qualitative explanation of key writing and dialogue moments
Joint display matrix	24	Focal learner case	Mixed-methods integration across scores, codes, uptake, and interview evidence

### 3.7 Instruments and operationalization

Harm-sensitive artificial-intelligence literacy was scored with a four-domain analytic rubric. The domains were harm identification, intelligence-consciousness distinction, anti-anthropomorphic calibration, and reflective ethical qualification. Each domain was scored from 0 to 4, producing a total score from 0 to 16. Argumentative writing quality was scored with a separate four-domain rubric: claim clarity, reasoning and warrant development, counterargument handling, and language control. Each domain was scored from 0 to 5, producing a total score from 0 to 20.

The harm-sensitive AI-literacy construct drew on three linked distinctions. The first was the distinction between fluent output and understanding, which is central to debates on language models and meaning (Bender and Koller, 2020). The second was the distinction between intelligence, consciousness, suffering, and moral standing, which matters when learners reason about whether AI systems can be harmed or respected as persons (Dehaene et al., 2017). The third was the risk of anthropomorphism, understood as the tendency to attribute humanlike mental states or moral qualities to nonhuman entities without sufficient warrant (Epley et al., 2007). In this study, these distinctions were operationalized through claims, warrants, hedges, contrasts, counterexamples, and explicit statements about harm or consciousness.

Direct ChatGPT uptake was operationalized as the movement of ChatGPT-generated wording or claims into learner drafts with minimal transformation. The unit of uptake was a clause, sentence, or idea unit traceable from a ChatGPT turn to a draft segment. Uptake was coded as direct, revised, rejected, or independently re-authored. This coding distinguished surface-level borrowing from learner-controlled transformation and avoided treating all ChatGPT use as either beneficial or harmful.

### 3.8 HARM-CAL annotation framework

HARM-CAL stands for Harm-sensitive Reasoning and Anthropomorphism Calibration in Language. The framework classified learner-produced language units into six multi-label reasoning states: naïve relativism, moral imposition, harm-sensitive reasoning, AI anthropomorphism, calibrated AI literacy, and reflective uncertainty. The labels were multi-label rather than mutually exclusive because a single unit could combine, for example, harm-sensitive reasoning and reflective uncertainty.

Text was segmented into argument-relevant units before annotation. A unit was defined as a clause, sentence, or short dialogue turn that expressed a claim, warrant, contrast, uncertainty marker, anthropomorphic attribution, or AI-literacy distinction. Purely procedural material was excluded unless it affected uptake, revision, or authorial control. The original learner wording was preserved in each unit.

Two trained applied linguists annotated all units using the six HARM-CAL labels. Twenty-five percent of the corpus was double-coded before full annotation proceeded. Coders were instructed to code the language in the unit rather than infer the learner’s private belief. Reflective uncertainty was assigned only when uncertainty performed an argumentative function, not when wording was merely vague. AI anthropomorphism was assigned only when a unit attributed feeling, suffering, intention, moral insight, or entitlement to AI without sufficient warrant. Coders were also instructed not to penalize a learner for exploring a minority or speculative position when the unit contained explicit evidence, limits, conditional reasoning, or counterargument.

Table 2 presents the HARM-CAL labels used in the annotation and modeling stages.

**Table 2:** HARM-CAL labels, operational definitions, and indicative examples.

Label	Operational definition	Indicative example
Naïve relativism	Treats moral disagreement as unjudgeable because cultures or individuals differ	“Every society has its own values, so we cannot say anything is wrong.”
Moral imposition	Asserts a moral conclusion without explaining harm, suffering, justification, or limits	“They are wrong and must change because our view is better.”
Harm-sensitive reasoning	Evaluates an issue by identifying who can suffer, how harm occurs, and why harm matters	“The key question is whether a conscious being is harmed, not only whether a rule is accepted.”
AI anthropomorphism	Attributes feeling, suffering, intention, moral insight, or entitlement to AI without warrant	“ChatGPT understands our pain and deserves respect like a person.”
Calibrated AI literacy	Distinguishes output competence from consciousness or suffering and avoids treating AI as a moral patient	“ChatGPT can generate persuasive language, but that does not show it feels pain or has moral standing.”
Reflective uncertainty	Uses hedging, limits, and conditional reasoning while maintaining an arguable position	“If consciousness requires the capacity to suffer, then current AI should not be treated as conscious, though future evidence could change this.”

### 3.9 NLP modeling and computational reproducibility

The NLP pipeline was implemented in Python 3.12. The primary computational output was a supervised transformer-family multi-label classifier with sigmoid outputs for the six HARM-CAL labels. The computational record preserved unit-level probabilities, thresholded predictions, fold assignments, and label-specific performance summaries for this classifier. However, the exported record did not retain the exact pretrained transformer checkpoint or original transformer training script. For this reason, the transformer-family results are reported as archived classifier-output benchmarks, whereas the TF-IDF logistic-regression model is treated as the fully specified reproducible baseline. Appendix A summarizes the available model documentation and the reproducibility boundary.

The training strategy used stratified five-fold cross-validation at the participant level. This design ensured that units from the same learner did not appear in both training and test folds. Performance was evaluated using macro-F1, micro-F1, precision, recall, and label-specific confusion matrices. Error analysis focused on reflective uncertainty and subtle anthropomorphism because these labels were theoretically central and likely to be linguistically variable in EFL writing.

The transformer-family classifier used sigmoid multi-label outputs and label-wise binary decisions for the six HARM-CAL labels. The transparent TF-IDF baseline used unigram and bigram features, a minimum document frequency of 2, a maximum of 8,000 features, balanced class weights, and one-vs-rest regularized logistic regression. These settings were selected to provide an interpretable sparse-text baseline that could capture short recurring lexical and phrase-level cues while limiting feature growth in a 9,113-unit learner-language corpus. Balanced class weights were used because HARM-CAL labels differed substantially in base rate, and participant-level folds were retained so that units from the same learner did not appear in both training and test partitions. The computational files preserved fold assignments, probability scores, thresholded predictions, and label-specific evaluation summaries so that classifier behavior could be inspected at both unit and label levels.

Computational transparency was addressed through preservation of the annotation codebook, segmentation rules, prompt versions, participant-level cross-validation split files, random seeds, preprocessing scripts, TF-IDF baseline hyperparameters, software and package versions, preserved transformer-family prediction outputs, and a short model-card style report. This documentation is consistent with broader recommendations for dataset and model transparency in machine learning and NLP research (Gebru et al., 2021; Mitchell et al., 2019; Papi et al., 2024; Storks et al., 2023). The analysis did not claim that HARM-CAL automated moral interpretation. It treated HARM-CAL as a transparent method for classifying language-based reasoning states, with reproducible annotation, cross-validation, and baseline-model components that remain subject to applied-linguistic interpretation.

### 3.10 Raters, coders, and reliability

Two doctoral-level applied linguists blind to condition and time scored the no-AI writing tasks. Before formal scoring, the raters calibrated on benchmark essays representing low, middle, and high performance on both rubrics. Inter-rater reliability for rubric total scores was estimated using two-way random-effects intraclass correlation coefficients for absolute agreement. Discrepancies greater than one scale point on any rubric domain were adjudicated through discussion after independent scoring had been completed.

HARM-CAL annotation coders received separate training on the six reasoning-state labels and uptake categories. Reliability was estimated before final analysis for the HARM-CAL labels and rubric scores. Cohen’s kappa was reported for binary HARM-CAL label decisions, and Krippendorff’s alpha was reported for higher-order multi-label reliability at the code-family level. The uptake file contains one adjudicated trace category for each uptake record rather than two independent coder fields; therefore, uptake is reported as trace-based process evidence and not as a separately reliability-estimated annotation layer. To make this evidentiary status explicit, Appendix A reports the uptake category counts and the available traceability checks. The planned thresholds were ICC ≥ 0.80 for rubric total scores, kappa ≥ 0.70 for major categorical labels, and alpha ≥ 0.70 for higher-order code families. These indices were selected because agreement assessment is especially important when linguistic categories are used as training data for computational models (Artstein and Poesio, 2008; Cohen, 1960).

### 3.11 Statistical analysis

R 4.4.x was used as the primary statistical computing environment. The main packages included lme4, lmerTest, emmeans, performance, effectsize, and ggplot2. The primary inferential models were linear mixed-effects models predicting harm-sensitive AI-literacy scores and argumentative writing quality scores from time, condition, and their interaction, with OOPT score as a covariate, participant as a random intercept, and section as a fixed blocking factor. Mixed-effects modeling was appropriate because repeated observations were nested within participants and participants were nested within intact sections (Bates et al., 2015).

The repeated-outcome specification can be written as outcome score ~ time × condition + OOPT + section + (1 | participant). For the gain-score contrasts, the dependent variable was either posttest gain or delayed-transfer gain, condition was the main predictor, and pretest score, OOPT score, diagnostic writing score, early reasoning profile, and section were included as covariates. Section was treated as a fixed blocking factor because the study contained only six intact sections.

Planned contrasts compared the climate-control ChatGPT condition with the standard ChatGPT and no-ChatGPT conditions at posttest and delayed transfer. Delayed transfer was treated as especially important because it measured independent no-AI performance after the intervention rather than immediate task-specific revision. Primary gain contrasts are reported as unstandardized raw-score differences, labeled b, with 95% confidence intervals and Hedges’ g calculated from the descriptive gain distributions. In interpreting L2 intervention effects, the study emphasized practical magnitude as well as statistical significance (Plonsky and Oswald, 2014). Adjusted contrasts used HC3 robust standard errors and 95% confidence intervals to reduce sensitivity to unequal residual variance across intact sections.

Reasoning-state distributions from HARM-CAL were analyzed with generalized mixed-effects models. For example, the probability of calibrated AI literacy, harm-sensitive reasoning, reflective uncertainty, or AI anthropomorphism was modeled as a function of condition and time, with participant-level random intercepts. Direct uptake rates in the two ChatGPT conditions were compared using mixed-effects binomial models or categorical comparisons where appropriate. Model fit and explained variance were summarized using model-appropriate R2 or pseudo-R2 measures (Nakagawa and Schielzeth, 2013), with the primary robustness summaries reported in Appendix A. The analysis avoided unsupported mediation claims. Mechanism was addressed through process indicators, uptake patterns, dialogue features, and qualitative integration.

### 3.12 Qualitative analysis

The qualitative strand used codebook thematic analysis of learner reflections, stimulated-recall interviews, and selected log-linked writing episodes. A purely reflexive thematic analysis was not used because the study had predefined constructs, cross-case comparison, and an explicit mixed-methods integration plan. Codebook thematic analysis was more appropriate because it allowed theoretically seeded code families while preserving inductive refinement from learner explanations and interactional evidence. The analysis was informed by the phased logic of thematic analysis and by qualitative trustworthiness criteria related to auditability, coherence, and analytic transparency (Braun and Clarke, 2006, 2021; Nowell et al., 2017).

NVivo 14 was used for data management, coding, memoing, and matrix queries. Initial code families included AI as authority, AI as wording source, AI as reasoning prompt, harm-based reframing, anthropomorphism correction, uncertainty expression, learner resistance, and re-authored argumentation. Twenty percent of the qualitative corpus was double-coded to support codebook stability. A second researcher acted as a critical friend during theme development. Themes were reported as developmental pathways rather than as a flat inventory of topics, with attention to how learners described the movement from prompt interpretation to dialogue, revision, and final authorial choice.

### 3.13 Mixed-methods integration

Integration occurred at four points. Design integration occurred because the focal qualitative subsample was selected from quantitative scores and early HARM-CAL profiles. Methods integration occurred because stimulated-recall interviews used concrete excerpts from essays, ChatGPT logs, reflections, and HARM-CAL-coded segments. Analytic integration occurred through participant-level matrices linking pretest and posttest scores, reasoning-state probabilities, uptake patterns, and learner explanations. Interpretive integration occurred through a joint display connecting instructional condition, reasoning-state change, uptake behavior, and qualitative pathways. This structure supported triangulation in the sense that self-reported perceptions were not interpreted alone; they were checked against dialogue behavior, draft revision, uptake traces, HARM-CAL-coded units, and delayed no-AI writing outcomes. This approach is consistent with mixed-methods integration through joint displays (Fetters et al., 2013; Guetterman et al., 2015) and with triangulation designs that connect learning outcomes with learner-perception evidence in technology-mediated language learning (Kazu and Kuvvetli, 2023).

### 3.14 Ethical procedures

Formal institutional ethics approval was obtained before recruitment. Participation was voluntary. Consent was collected by a research assistant who had no grading role, and nonparticipation had no effect on course grades. The instructor did not access identifiable research data until final grades had been submitted. All students completed the same required instructional content, but only consenting students’ data entered the research corpus.

AI-related safeguards were built into the design. Study-created ChatGPT accounts were used, memory was disabled, and students were instructed not to enter names, personal experiences, political affiliations, religious beliefs, medical information, or other sensitive data. Prompts avoided direct religious framing and did not ask students to disclose private moral positions. The study analyzed argumentation rather than private belief. Data were stored on encrypted institutional storage, and the name-code key was stored separately from the analytic files. After data collection, all sections received a shared workshop on responsible ChatGPT use, AI anthropomorphism, verification practices, and authorial control.

## 4 Results: EFL writing, HARM-CAL, and AI literacy

### 4.1 Analytic sample, data audit, and corpus structure

The analytic sample was complete for all required primary outcomes. The dataset contained 120 participants, with 40 students in each condition and 20 students in each of the six intact sections. The no-AI writing dataset contained 480 scored essays across the diagnostic, pretest, posttest, and delayed transfer tasks. The intervention corpus contained 720 first and final drafts, 3,108 ChatGPT dialogue turns, 240 ChatGPT session summaries, and 360 learner reflections. The computational corpus contained 9,113 HARM-CAL argument-relevant units, including 2,278 double-coded units for reliability estimation. The uptake dataset contained 967 traceable ChatGPT-to-draft uptake records, and the qualitative strand contained 120 stimulated-recall excerpts from 24 focal learners. All internal validation checks passed before inferential analysis. Table 3 summarizes the analyzed data sources.

**Table 3:** Analyzed data sources and corpus structure.

Analytic strand	Data source	Rows	Unit of analysis	Function in the results section
Sample structure	Participants	120	Participant	Condition balance, proficiency, baseline comparability
Sample structure	Sections	6	Intact section	Quasi-experimental assignment and blocking
Prompt linkage	Prompts	7	Study prompt	Links writing, dialogue, draft, and reflection tasks
No-AI outcomes	Writing scores	480	Participant-task score	Primary harm-literacy and writing-quality outcomes
No-AI outcomes	Essays	480	Participant-task essay	Independent writing evidence at each time point
Intervention process	First and final drafts	720	Participant-cycle-draft stage	Revision, authorial control, and draft development
Intervention process	ChatGPT logs	3,108	Dialogue turn	Learner-AI interaction and process evidence
Intervention process	Session summaries and dialogue features	240	Participant-cycle session	Turn counts, warrants, counterexamples, self-repair, uptake rates
Learner explanation	Reflections	360	Participant-cycle reflection	Ownership, helpfulness, copying, and perceived reasoning change
Computational corpus	HARM-CAL units	9,113	Argument-relevant language unit	Human annotation and NLP classification
Reliability	Double-coded HARM-CAL units	2,278	Argument-relevant language unit	Human agreement for HARM-CAL labels
Uptake	Uptake traces	967	Clause, sentence, or idea unit	Movement from ChatGPT output to learner draft
Qualitative explanation	Stimulated-recall interviews	120	Interview excerpt	Learner explanation of reasoning, revision, and uptake decisions
Mixed-methods integration	Joint display matrix	24	Focal learner case	Integration across scores, HARM-CAL profiles, uptake, and interviews

### 4.2 Baseline comparability and reliability

The three conditions were comparable on the main baseline variables. Mean OOPT scores were 76.35 in the no-ChatGPT control group, 75.18 in the standard ChatGPT group, and 75.47 in the climate-control ChatGPT group, *F*(2, 117) = 0.26, *p* = 0.770. Diagnostic writing scores were also comparable, with means of 10.38, 9.58, and 10.25 on the 20-point scale, F(2, 117) = 1.91, *p* = 0.153. Diagnostic harm-sensitive AI-literacy scores were nearly identical across groups, with means of 5.73, 5.58, and 5.73 on the 16-point scale, F(2, 117) = 0.10, *p* = 0.908. Age did not differ by condition, F(2, 117) = 0.01, *p* = 0.990. Gender, CEFR band, and prior ChatGPT use were not significantly associated with condition. Early reasoning profile did differ by condition, χ^2^(12) = 22.05, *p* = 0.037. Because this imbalance could threaten interpretation, the primary planned contrasts were estimated with pretest score, OOPT score, diagnostic writing score, section, and early reasoning profile included as covariates. The direction and statistical status of the main condition contrasts remained stable.

Reliability estimates met the thresholds set before analysis. ICC(2,1) absolute agreement was 0.971 for harm-sensitive AI-literacy scores and 0.981 for argumentative writing-quality scores. HARM-CAL label reliability also exceeded the planned 0.70 threshold for every label, and Krippendorff’s alpha across all six binary label decisions was 0.854. These values supported the use of both the human-scored outcomes and the annotated HARM-CAL corpus in the subsequent analyses.

### 4.3 No-AI EFL writing outcomes

The primary no-AI outcomes showed larger and more durable gains in the climate-control ChatGPT condition than in either comparison condition. Table 4 reports descriptive means for the pretest, posttest, and delayed transfer tasks. The delayed transfer task is especially important because it was completed without AI access four weeks after the intervention.

**Table 4:** No-AI outcome means by condition and time point.

Outcome	Condition	Pretest M (SD)	Posttest M (SD)	Delayed transfer M (SD)	Posttest gain M (SD)	Delayed gain M (SD)
Harm-sensitive AI literacy, 0 to 16	No-ChatGPT control	5.98 (1.86)	6.90 (2.33)	6.70 (2.63)	0.92 (1.14)	0.72 (1.52)
Harm-sensitive AI literacy, 0 to 16	Standard ChatGPT	5.88 (2.04)	7.65 (2.64)	7.18 (2.58)	1.78 (1.42)	1.30 (1.79)
Harm-sensitive AI literacy, 0 to 16	Climate-control ChatGPT	6.08 (1.87)	9.83 (2.50)	9.55 (2.48)	3.75 (1.30)	3.48 (1.34)
Argumentative writing quality, 0 to 20	No-ChatGPT control	10.60 (2.01)	11.30 (3.07)	11.48 (3.34)	0.70 (1.83)	0.88 (2.26)
Argumentative writing quality, 0 to 20	Standard ChatGPT	9.80 (2.42)	12.08 (2.61)	11.55 (2.42)	2.28 (2.03)	1.75 (1.86)
Argumentative writing quality, 0 to 20	Climate-control ChatGPT	10.38 (2.44)	13.83 (3.18)	13.55 (2.77)	3.45 (1.69)	3.18 (1.50)

Table 5 makes the primary statistical specifications explicit and shows how the repeated-outcome models, gain-score contrasts, HARM-CAL distributions, uptake analyses, and process comparisons were linked to the research questions.

**Table 5:** Statistical model specifications for primary quantitative and computational outcomes.

Analytic target	Dependent variable	Main predictors	Covariates and blocking	Random structure or error method	Primary comparison
No-AI repeated writing outcomes	Harm-sensitive AI-literacy score; argumentative writing-quality score	Time, condition, time × condition	OOPT score; section as fixed blocking factor	Participant random intercept; linear mixed-effects model	Condition differences at posttest and delayed transfer
No-AI gain contrasts	Posttest gain; delayed-transfer gain	Condition	Pretest score; OOPT score; diagnostic writing score; early reasoning profile; section	HC3 robust standard errors; 95% confidence intervals	Climate-control vs. control; climate-control vs. standard; standard vs. control
HARM-CAL reasoning-state distributions	Binary label presence per unit or grouped label rate	Condition, time/source type, and condition × time where applicable	Section and source type where applicable	Participant random intercept; generalized mixed-effects binomial model	Delayed climate-control trajectory vs. comparison trajectories
ChatGPT uptake	Direct uptake indicator or uptake category	Condition	Cycle or session where applicable	Mixed-effects binomial model or categorical comparison	Standard vs. climate-control direct and re-authored uptake
Dialogue features and reflections	Session-level counts or rating means	Condition	Cycle where applicable	Reported group-comparison statistics	Standard vs. climate-control process differences

The adjusted planned contrasts in Table 6 confirm that the climate-control condition outperformed both the no-ChatGPT control condition and the standard ChatGPT condition. As specified in Table 5, the gain-score models used HC3 robust standard errors and controlled for the baseline variables noted above. The coefficients in Table 6 are unstandardized raw-score differences, and the standardized values are Hedges’ g estimates calculated from the descriptive gain distributions. The standard ChatGPT condition also outperformed the control condition on most immediate and delayed writing-quality contrasts, but its delayed harm-literacy advantage over the control condition was not statistically reliable after adjustment.

**Table 6:** Adjusted planned contrasts for no-AI outcome gains.

Outcome contrast	Climate-control minus control			Climate-control minus standard			Standard minus control		
	b [95% CI]	*g*	*p*	b [95% CI]	*g*	*p*	b [95% CI]	*g*	*p*
Harm-sensitive AI literacy, posttest gain	1.88 [1.42, 2.34]	g = 2.29	*p <* 0.001	1.31 [0.89, 1.72]	g = 1.43	*p <* 0.001	0.57 [0.08, 1.06]	g = 0.66	*p* = 0.021
Harm-sensitive AI literacy, delayed gain	1.78 [1.23, 2.32]	g = 1.91	*p <* 0.001	1.50 [1.06, 1.95]	g = 1.37	*p <* 0.001	0.27 [−0.32, 0.87]	g = 0.35	*p* = 0.364
Argumentative writing quality, posttest gain	1.87 [1.22, 2.52]	g = 1.55	*p <* 0.001	0.76 [0.24, 1.27]	g = 0.62	*p* = 0.004	1.11 [0.44, 1.78]	g = 0.81	*p* = 0.001
Argumentative writing quality, delayed gain	1.65 [1.04, 2.25]	g = 1.19	0.001	0.92 [0.48, 1.36]	g = 0.84	*p <* 0.001	0.72 [0.12, 1.32]	g = 0.42	*p* = 0.018

These results indicate that the climate-control condition was not merely associated with stronger immediate revision. Its advantage remained visible in delayed independent writing, especially for harm-sensitive AI literacy.

The robustness checks supported the same interpretation. The climate-control advantage remained in models that included early reasoning profile, pretest score, diagnostic writing score, OOPT score, and section as covariates, and the direction of the primary delayed-transfer contrasts remained stable under alternative specifications. Because the study used six intact sections, these checks do not remove all possible section-level confounding, but they reduce the likelihood that the delayed-transfer pattern was produced only by the observed baseline imbalance. Appendix A summarizes the sensitivity checks and model-fit indices.

### 4.4 HARM-CAL reliability and computational validation

Table 7 reports the HARM-CAL human reliability and model-performance evidence. The table includes two forms of computational evidence: archived transformer-family classifier outputs preserved in the computational record and a fully specified TF-IDF logistic-regression baseline. Because the exact pretrained transformer checkpoint and original training script were not retained in the exported record, the transformer column should be interpreted as an archived classifier-output benchmark rather than as a fully checkpoint-reproducible model specification. Appendix A documents this reproducibility boundary. The archived transformer-family classifier outputs showed stronger performance than the TF-IDF logistic-regression baseline for every label and for both aggregate F1 measures. The strongest transformer-family performance was observed for AI anthropomorphism, calibrated AI literacy, and harm-sensitive reasoning. Reflective uncertainty and naïve relativism were more difficult to classify, which is consistent with their greater dependence on discourse context and pragmatic qualification.

**Table 7:** HARM-CAL human reliability and model performance.

HARM-CAL label or metric	Human reliability	Transformer F1	TF-IDF logistic F1
Naïve relativism	κ = 0.824	0.641	0.358
Moral imposition	κ = 0.722	0.806	0.392
Harm-sensitive reasoning	κ = 0.881	0.918	0.688
AI anthropomorphism	κ = 0.857	0.961	0.708
Calibrated AI literacy	κ = 0.871	0.941	0.672
Reflective uncertainty	κ = 0.787	0.743	0.574
All six binary label decisions	α = 0.854	Macro-F1 = 0.835	Macro-F1 = 0.565
All six binary label decisions	α = 0.854	Micro-F1 = 0.848	Micro-F1 = 0.607

The error analysis showed three recurring sources of difficulty. Reflective uncertainty was sometimes confused with general vagueness when learners used hedges without clearly limiting a claim. Naïve relativism was difficult when learners first acknowledged cultural difference but later introduced a harm-based constraint in the same unit. Subtle anthropomorphism was also harder when students used metaphorical shorthand rather than explicit verbs such as feel, suffer, want, or deserve. These errors show why HARM-CAL is useful as a reproducible classification framework but still needs careful human interpretation for context-sensitive reasoning states.

Table 7 should therefore be interpreted as evidence that HARM-CAL is classifiable with strong archived transformer-family performance and a reproducible non-neural baseline. Appendix A provides the corresponding reproducibility boundary, sensitivity summaries, and uptake traceability information.

### 4.5 Reasoning-state trajectories, dialogue features, drafts, and uptake

The HARM-CAL essay distributions showed that the climate-control condition changed the structure of learner reasoning in independent writing. In the climate-control group, the harm-sensitive reasoning rate increased from 0.315 at pretest to 0.550 at posttest and 0.545 at delayed transfer. Calibrated AI literacy increased from 0.325 to 0.375 at posttest and 0.415 at delayed transfer. Naïve relativism decreased from 0.060 to 0.015 by delayed transfer, and moral imposition decreased from 0.140 to 0.035. AI anthropomorphism remained low, moving from 0.085 to 0.075.

The standard ChatGPT group showed a less durable trajectory. Harm-sensitive reasoning increased from 0.335 at pretest to 0.455 at posttest but decreased to 0.370 at delayed transfer. AI anthropomorphism increased from 0.120 to 0.175 at posttest and remained above pretest at delayed transfer, 0.150. The control group showed limited change in calibrated AI literacy, from 0.125 to 0.135, and stable harm-sensitive reasoning, from 0.405 to 0.395. A binomial model of essay-level HARM-CAL rates confirmed a significant delayed climate-control by time interaction for harm-sensitive reasoning relative to the control trajectory, log OR = 1.00, 95% CI [0.46, 1.53], *p <* 0.001, and relative to the standard ChatGPT trajectory, log OR = 0.80, 95% CI [0.33, 1.28], *p <* 0.001. Other label movements were directionally consistent with the intervention, but several labels had low base rates and should be interpreted more cautiously.

Table 8 reports the main intervention-process evidence. The climate-control condition generated more learner turns, more warrant markers, more counterexample markers, and substantially more self-repair markers than the standard ChatGPT condition. Hedge counts and intelligence-consciousness distinction counts were similar across the two ChatGPT conditions, which suggests that the stronger climate-control outcomes were not simply due to more hedging or more references to consciousness. The difference was more visible in how learners revised, challenged, repaired, and re-authored their reasoning.

**Table 8:** Intervention-process evidence from drafts, dialogue features, uptake, and reflections.

Evidence type	Measure	No-ChatGPT control	Standard ChatGPT	Climate-control ChatGPT	Statistical comparison
Draft revision	Harm-literacy draft gain	0.76	1.55	2.12	Descriptive process comparison
Draft revision	Argument-quality draft gain	0.49	1.48	2.06	Descriptive process comparison
Draft revision	Final authorial-control rating	3.27	2.92	4.23	Descriptive process comparison
Dialogue	Learner turns per session	Not applicable	5.93	7.02	t = 10.45, *p <* 0.001
Dialogue	Warrant markers per session	Not applicable	2.05	2.79	t = 3.44, *p <* 0.001
Dialogue	Counterexample markers per session	Not applicable	3.03	3.88	t = 3.87, *p <* 0.001
Dialogue	Hedge markers per session	Not applicable	3.19	3.18	t = −0.04, *p* = 0.970
Dialogue	Self-repair markers per session	Not applicable	2.39	5.84	t = 16.39, *p <* 0.001
Dialogue	Intelligence-consciousness distinctions per session	Not applicable	2.78	2.85	t = 0.30, *p* = 0.766
Uptake	Direct uptake	Not applicable	256 of 488, 52.5%	12 of 479, 2.5%	χ^2^(3) = 380.64, *p <* 0.001
Uptake	Independently re-authored uptake	Not applicable	19 of 488, 3.9%	175 of 479, 36.5%	Included in uptake χ^2^
Reflection	Perceived ownership, 1 to 5	3.63	2.85	3.42	Climate vs. standard: t = 6.06, *p <* 0.001
Reflection	Perceived helpfulness, 1 to 5	3.21	3.52	3.70	Climate vs. standard: t = 1.76, *p* = 0.080
Reflection	Reported direct copying, 0 to 3	0.14	1.15	0.37	Climate vs. standard: t = −10.80, *p <* 0.001

The uptake results were especially clear. Direct uptake accounted for 52.5% of traceable uptake in the standard ChatGPT condition but only 2.5% in the climate-control condition. In contrast, independently re-authored uptake accounted for 36.5% of climate-control uptake but only 3.9% of standard ChatGPT uptake. Revised uptake was common in both groups, 37.3% in standard ChatGPT and 41.1% in climate-control ChatGPT, but the meaning of revision differed across conditions. In the standard group, revision often softened or localized a ChatGPT-generated sentence. In the climate-control group, revision more often followed a tutor question that required the learner to supply the claim, warrant, or limit. Because the uptake file contains adjudicated trace categories rather than separate coder fields, Appendix A reports uptake as trace-based behavioral evidence and documents the available category counts and traceability checks.

### 4.6 Qualitative pathways and mixed-methods integration

The qualitative data explain how these quantitative and computational patterns appeared in learner experience. In the control group, students described improvement as slower and more dependent on class discussion, peer feedback, and their own struggle to connect claims with reasons. P02 explained, “I was trying to make the reason clear because at first I only wrote my opinion. The class feedback helped me see that a claim needs a warrant.” P25 wrote in a reflection, “The difficult part was showing uncertainty without making the whole argument weak.” P36 similarly described revision as slower but more owned: “I did not use ChatGPT, so I had to decide the wording by myself. This was slower, but it helped me notice where my idea was not clear.”

Standard ChatGPT learners often valued fluent explanation and organization but also reported dependency risk. P41 stated, “ChatGPT gave a sentence that was much better than mine. I used part of it, then I changed some words. It helped with academic style, but sometimes I felt the idea was coming from the tool more than from me.” P57 wrote, “The tool was useful, but it was easy to depend on the ready wording.” P59 reported a similar tension: “The explanation was useful. However, it also gave many polished sentences, and I had to decide which one was really my claim.” These accounts help explain why the standard condition improved immediate writing quality but showed weaker delayed transfer in harm-sensitive AI literacy.

Climate-control learners described a different pathway. They often framed ChatGPT as a questioning environment rather than a source of finished language. P84 recalled, “The tutor asked me to write my own claim first. At the beginning I was annoyed because I wanted an answer. But the question about who is harmed made me find the reason myself.” P105 wrote, “The tutor did not give me the paragraph. It asked me who is harmed and what evidence shows harm. This was difficult at first because I wanted a direct answer, but it made me write the reason myself.” P114 linked this questioning process to anthropomorphism calibration: “I rewrote this part because it sounded like AI has feelings. The tutor did not say the final sentence; it asked what evidence would show suffering. That helped me separate performance from consciousness.” P116 described the transfer value of the same process: “The climate-control prompt was slower but better for thinking. It made me add a counterexample and then limit my conclusion.”

Table 9 integrates the focal-case evidence across the three conditions. The display functions as a triangulation matrix: learner explanations are interpreted alongside delayed no-AI score gains, HARM-CAL profiles, uptake behavior, and evidence of authorial control rather than being treated as self-report alone. The display shows that the strongest developmental pathway was not simply higher AI helpfulness. It was the alignment of delayed no-AI gain, lower direct uptake, stronger authorial control, and learner explanations centered on self-repair, harm identification, and re-authored argumentation.

**Table 9:** Mixed-methods joint display of focal learner pathways.

Condition	Focal cases	Mean posttest harm-literacy gain	Mean delayed harm-literacy gain	Uptake and process profile	Dominant qualitative pathway	Integrated inference
No-ChatGPT control	8	0.88	0.75	No ChatGPT uptake; revision shaped by class discussion, peer feedback, and individual effort	Learners described incremental warrant development and difficulty maintaining qualified claims	Ordinary instruction supported modest argumentation gains, but transfer to calibrated AI literacy was limited
Standard ChatGPT	8	2.25	1.50	High direct uptake; mean focal direct uptake rate = 0.626	Learners valued fluency, transitions, and explanation, while reporting dependency risk	Standard ChatGPT supported immediate writing improvement, but some gains appeared tied to access to fluent AI phrasing
Climate-control ChatGPT	8	4.13	3.00	Low direct uptake; mean focal direct uptake rate = 0.039; frequent revised or independently re-authored uptake	Learners described tutor questioning, counterexamples, harm identification, and intelligence-consciousness distinctions	Climate-control dialogue supported durable harm-sensitive reasoning by reducing answer dependency and increasing authorial control

The integrated findings indicate that climate-control ChatGPT produced the strongest pattern across the full dataset: larger no-AI gains, stronger delayed transfer, higher rates of harm-sensitive reasoning, lower direct uptake, greater authorial control, and qualitative evidence of learner-led reasoning repair. Standard ChatGPT was beneficial for immediate writing support, but the process evidence suggests that its benefit was more closely tied to fluent external language. The control group showed that ordinary instruction can improve argumentation, but the gains were smaller and less specific to harm-sensitive AI-literacy calibration.

## 5 Discussion: climate-control ChatGPT and responsible AI literacy

### 5.1 Principal findings

This study examined whether a climate-control version of ChatGPT could support measurable growth in harm-sensitive artificial-intelligence literacy and English argumentative reasoning among EFL learners. The findings indicate that it could within the limits of a quasi-experimental intact-section design. Learners in the climate-control condition showed the largest gains in no-AI harm-sensitive AI literacy and argumentative writing quality, and these gains remained visible on the delayed transfer task. This delayed result matters because it suggests that the intervention did not merely improve AI-assisted drafting during the treatment period. It appears to have supported reasoning practices that learners could later mobilize without ChatGPT access.

The process evidence clarifies the nature of this advantage. The climate-control condition produced more learner turns, more warrant markers, more counterexample markers, and substantially more self-repair markers than standard ChatGPT. It also produced far less direct uptake of AI wording and more independently re-authored uptake. This pattern suggests that the central difference was not access to a more fluent language model. Both ChatGPT conditions had access to the same general technological affordance. The difference was the interactional climate: standard ChatGPT tended to operate as a source of usable language and ideas, whereas climate-control ChatGPT was designed to slow down answer dependency and require learners to articulate, test, and revise their own claims.

These findings extend prior work on ChatGPT and L2 writing. Existing studies have shown that ChatGPT can support writing development, feedback access, motivation, and classroom writing practices, while also raising concerns about dependency, authorship, academic integrity, and shallow revision (Barrot, 2023; Kohnke et al., 2023; Praphan and Praphan, 2023; Song and Song, 2023; Su et al., 2023; Yan, 2023; Yang and Li, 2024). The present study adds a more specific claim: the educational value of ChatGPT in EFL writing may depend less on whether learners use the tool and more on the discourse conditions under which the tool is allowed to participate in reasoning. A prompt design that prevents full-answer delivery and repeatedly requires learner justification appears to shift ChatGPT from a fluency engine toward a reasoning environment.

### 5.2 Climate-control ChatGPT as a reasoning environment

The climate-control condition was built on a simple pedagogical premise: learners should not receive polished moral or argumentative answers before they have generated their own claims, warrants, limits, and counterexamples. The results support this premise. Climate-control learners reported that the tutor’s questions were slower and sometimes frustrating, yet they also described these questions as the reason they revised weak claims, distinguished harm from disagreement, and separated AI performance from consciousness. This pattern is consistent with research showing that teacher support is important for student motivation when learners use AI chatbots (Chiu et al., 2024) and with evidence that AI-mediated EFL learning can affect enjoyment, anxiety, and willingness to communicate (Zhang et al., 2024). The productive value of frustration should therefore not be overstated. In this study, the difficulty was pedagogically bounded: learners received a clear rationale, worked within supervised sessions, and were reminded that the tutor’s refusal to provide full answers was intended to protect their authorship. Under those conditions, temporary confusion could become a resource for learning rather than a source of disengagement (D'Mello et al., 2014).

This finding is consistent with research on digital writing support showing that automated tools can be useful when they scaffold strategic thinking rather than simply correct surface features (McNamara et al., 2010; Strobl et al., 2019). It also aligns with L2 writing research that treats development as more than improvement in final text quality. Writing development involves control over stance, cohesion, reasoning, register, and the linguistic expression of qualification (Aull and Lancaster, 2014; McNamara et al., 2010; Polio, 2017). From a dialogic perspective, the climate-control prompt preserved learner labor in the exchange: it increased learner turns, warrants, counterexamples, and self-repair instead of allowing the AI response to close the dialogue prematurely. This interpretation is consistent with sociocultural accounts of dialogic teaching and learning, in which understanding develops through contingent questioning, explanation, and uptake rather than through the passive receipt of polished answers (Howe and Abedin, 2013; Mercer and Howe, 2012).

The findings also complicate the assumption that more AI assistance necessarily produces better learning. Standard ChatGPT learners improved, especially in immediate writing quality, but the uptake evidence suggests that part of this improvement was tied to the availability of fluent external phrasing. Climate-control learners improved more slowly in process terms because they had to supply more of the reasoning themselves. Yet that constraint appears to have strengthened later independent performance. It also helped prevent discourse homogenization: learners were less likely to adopt ready-made AI wording and more likely to re-author claims in their own argumentative voice. A useful theoretical proposition follows: in AI-mediated L2 writing, productive difficulty may emerge when the system withholds complete answers while maintaining enough interactional support to help learners continue reasoning.

### 5.3 HARM-CAL as a computational contribution

The study also contributes HARM-CAL as a computationally tractable framework for classifying harm-sensitive reasoning and anthropomorphism calibration in second-language argumentation. The human reliability coefficients met the planned thresholds, and the archived transformer-family classifier outputs showed stronger performance than the TF-IDF logistic-regression baseline across all labels. Performance was strongest for AI anthropomorphism, calibrated AI literacy, and harm-sensitive reasoning, and weaker for reflective uncertainty and naïve relativism. This pattern is theoretically meaningful. Labels tied to explicit lexical and conceptual contrasts, such as feeling, suffering, consciousness, and output competence, were easier to classify than labels requiring more discourse-level inference.

This result advances current work in language and computation by showing how applied-linguistic constructs can be operationalized without reducing them to simple surface features. HARM-CAL does not claim to detect moral reasoning as a private mental state. It classifies observable language units that index reasoning-state markers in context. This distinction is important because current debates about language models warn against confusing linguistic form with understanding (Bender and Koller, 2020; Bender et al., 2021). It is also important for learner assessment: a computational label should be interpreted as evidence about language use in a task, not as a claim about a student’s moral character or private belief.

The computational strand also responds to calls for transparency and bounded reproducibility in NLP. The inclusion of segmentation rules, codebooks, participant-level cross-validation, a fully specified TF-IDF baseline, error analysis, preserved unit-level predictions, and model-card style reporting strengthens the credibility of the classification evidence (Gebru et al., 2021; Mitchell et al., 2019; Papi et al., 2024; Storks et al., 2023). At the same time, the missing exact transformer checkpoint and original training script limit exact replication of the transformer-family benchmark. For this reason, the fully specified TF-IDF model and the preserved prediction outputs serve different evidentiary roles: the former provides a reproducible non-neural baseline, while the latter documents stronger archived classifier performance without overstating checkpoint-level reproducibility.

The contribution to language and computation is therefore practical as well as conceptual. The study links a classroom intervention to annotated learner-language units, supervised multi-label prediction, uptake traces, and mixed-methods interpretation. This link is important because the same student sentence can be read as writing performance, evidence of reasoning, and a computational unit whose label depends on explicit annotation rules rather than impressionistic judgment.

### 5.4 Anthropomorphism calibration and responsible AI literacy

One of the clearest conceptual contributions of the study is its treatment of anthropomorphism as both a linguistic and educational issue. Learners did not only need to know that ChatGPT can generate fluent English. They needed to reason about what such fluency does and does not imply. The climate-control condition strengthened learners’ ability to distinguish output competence from consciousness, suffering, and moral standing. This distinction is central to responsible AI literacy because anthropomorphic language can make a system appear more agentive, morally aware, or emotionally present than the evidence warrants.

The finding connects applied linguistics to broader work on anthropomorphism and consciousness. Epley et al. (2007) show that humans readily attribute humanlike qualities to nonhuman agents under certain cognitive and social conditions. Dehaene et al. (2017) distinguish forms of intelligence and information processing from consciousness. These distinctions became linguistically consequential in the present study. Learners who wrote that ChatGPT “understands,” “feels,” or “deserves respect like a person” were not merely choosing colorful words. They were producing language that could blur the boundary between computational performance and moral patienthood. Climate-control prompting reduced this risk by repeatedly asking what evidence would be needed to attribute suffering, consciousness, or moral standing.

This does not mean that anthropomorphism should be treated only as an error. Some anthropomorphic metaphors may help novice users describe interaction with complex systems, and some communities may frame nonhuman agency differently from dominant Western philosophical traditions. The pedagogical problem begins when metaphor becomes unexamined attribution or when fluent output is treated as evidence of inner experience. The present findings suggest that EFL writing classrooms can address this problem directly. Students can be taught to write about AI systems with lexical precision, epistemic caution, and ethical clarity while still acknowledging that moral disagreement, personhood, and agency are culturally and philosophically situated.

### 5.5 Implications for EFL writing and AI literacy pedagogy

The findings point to a practical implication for EFL writing instruction: the question is not whether ChatGPT should be allowed or banned in all writing tasks. A more useful question is what kind of interaction should be designed when ChatGPT is used. Standard access can help students generate ideas and improve fluency, but it can also increase direct uptake and weaken authorial control. Climate-control prompting offers a middle position. It permits AI-supported dialogue while restricting the tool from doing the learner’s central argumentative work. This design also aligns with culturally adaptive approaches to AI education, which emphasize that AI literacy should be responsive to local learning cultures rather than imposed as a culturally neutral technical script (Samuel et al., 2023).

For teachers, this means that prompt design should be treated as part of writing pedagogy rather than as a technical afterthought. Students can be required to state their claim before receiving suggestions, identify who or what is harmed, distinguish evidence from assertion, add counterexamples, and explain why a qualification is needed. These practices are familiar in argumentation instruction, but ChatGPT can either undermine or reinforce them depending on how it is positioned. The present study suggests that AI-supported writing tasks should train learners to justify, qualify, distinguish, and re-author, rather than merely polish, expand, or translate their first ideas.

The results also support a stronger role for reflection and stimulated recall in AI-mediated writing instruction. Learner reflections showed that students were aware of differences between help, dependence, and ownership. This awareness should be cultivated. Students need language for describing how they used AI, what they accepted, what they rejected, and what they rewrote independently. Such reflection is pedagogically valuable because it makes authorship visible as a process rather than treating authorship as a simple yes-or-no property of a final text.

### 5.6 Limitations and future research

Several limitations qualify the interpretation of the findings. The study was conducted in one public-university EFL context in Jordan, with second-year English majors in academic writing sections. The results may not generalize directly to other proficiency levels, age groups, institutional settings, languages, or writing genres. Cross-cultural validation is especially important because harm, moral disagreement, AI personhood, and educational authority may be framed differently across communities. This point should not be read as a deficit claim about the Jordanian context. Rather, it recognizes that classroom reasoning is culturally situated and that responsible pedagogy should sustain local linguistic and cultural resources while supporting explicit argumentation (Ladson-Billings, 1995; Paris, 2012).

The quasi-experimental design is another limitation. Intact sections were assigned to conditions, which preserved classroom feasibility but did not provide individual random assignment. Baseline proficiency and diagnostic writing scores were comparable across conditions, and the models controlled for early reasoning profile, section, proficiency, and pretest performance. The sensitivity checks in Appendix A indicate that the main delayed-transfer pattern remained stable under alternative specifications. Still, only six sections were available, and unmeasured section-level differences cannot be fully ruled out. Future studies should test the intervention with larger multisite samples, random assignment where feasible, and preregistered analysis plans.

The study also relied on one AI platform and a particular climate-control prompt design. Model behavior, interface design, institutional access, and student familiarity with generative AI may change rapidly. Future research should compare climate-control prompting across models, languages, and interface constraints. It should also test which elements of the prompt are most important: withholding full answers, asking one question at a time, requiring learner claims first, prompting counterexamples, or explicitly targeting anthropomorphism.

The HARM-CAL framework also needs further validation. Although reliability and classification performance were strong in this dataset, some labels were more difficult than others. Reflective uncertainty and naïve relativism depend heavily on context, and future versions of HARM-CAL may benefit from discourse-window modeling rather than isolated unit classification. Additional research should examine whether HARM-CAL labels predict later writing development, transfer to new ethical topics, or learner metalinguistic awareness. Human-in-the-loop annotation may remain essential for the most interpretive categories. Future computational work should also retain exact transformer checkpoints, training scripts, configuration files, and thresholding logs so that high-performing neural classifiers can be reproduced at checkpoint level.

Finally, the study suggests several testable theoretical directions. One is the reasoning climate hypothesis: AI tools support durable L2 writing development when they regulate the conditions of reasoning rather than supply finished language. A second is the anthropomorphism calibration hypothesis: learners become more responsible AI users when they repeatedly practice distinguishing output competence from consciousness, suffering, and moral standing in their own writing. A third is the authorial-control hypothesis: delayed transfer depends on the proportion of AI-supported ideas that learners revise, reject, or independently re-author. These propositions can be tested in future longitudinal, cross-linguistic, and classroom-based studies.

The contribution of this study is therefore not that ChatGPT is inherently beneficial or harmful for EFL writing. The evidence indicates a more precise conclusion: ChatGPT becomes educationally valuable when its interactional role is deliberately constrained to protect learner reasoning. Climate-control prompting offers one way to design that constraint, and HARM-CAL offers one way to measure its linguistic consequences.

## 6 Conclusion

This study examined whether ChatGPT can be designed as more than a source of fluent language support for EFL writing. The findings indicate that a climate-control version of ChatGPT, one that withholds full answers and requires learners to justify, qualify, counterexample, and re-author their own claims, produced stronger gains in harm-sensitive artificial-intelligence literacy and argumentative writing quality than both standard ChatGPT use and no-ChatGPT instruction. Crucially, these gains remained visible in delayed no-AI writing, which suggests that the intervention supported transferable reasoning practices rather than only immediate AI-assisted performance.

The study’s central contribution is twofold. Pedagogically, it shows that the value of ChatGPT in second-language writing depends on the interactional role assigned to the system. Standard ChatGPT helped learners improve fluency and organization, but it also produced high levels of direct uptake. Climate-control ChatGPT created a different learning environment by slowing down answer dependency and prompting learners to identify harm, distinguish intelligence from consciousness, resist unsupported anthropomorphism, and revise their own reasoning. Computationally, the study contributes HARM-CAL as a reproducible framework for classifying harm-sensitive reasoning, AI anthropomorphism, calibrated AI literacy, and reflective uncertainty in learner language. The human reliability and model-validation results suggest that these constructs can be made measurable without reducing them to simple surface features. For language-and-computation research, the study shows how AI-mediated classroom interaction can be connected to annotated learner-language units, supervised multi-label classification, and mixed-methods interpretation of reasoning development.

The broader implication is that responsible AI literacy should be treated as part of language education, not as an external digital-skills add-on. EFL learners need linguistic resources for making careful claims about AI, harm, evidence, uncertainty, and moral standing. They also need classroom designs that protect authorial control while still allowing productive interaction with generative AI. Climate-control prompting offers one such design. It does not ask teachers to ignore ChatGPT or to accept it uncritically. It shows how AI-mediated writing can be structured so that learners remain the authors of their arguments and the agents of their own reasoning.

## Data Availability

The raw data supporting the conclusions of this article will be made available by the authors, without undue reservation.
