# Supplementary material for: Climate-control ChatGPT for EFL writing and AI literacy
Source: Front Artif Intell. 2026 Jul 22;9:1869343. doi: 10.3389/frai.2026.1869343 (PMC13437626; doi:10.3389/frai.2026.1869343)
Supplement: Supplementary file 1 [file Supplementary_file_1.docx]

**Appendix A. Robustness, model documentation, and uptake traceability**

Appendix A summarizes supplementary robustness, documentation, and traceability checks that support interpretation of the primary analyses. It documents sensitivity checks for the gain-score models, the status of HARM-CAL computational transparency and bounded reproducibility, and the trace-based evidence used for uptake analysis.

**Table A1. Robustness checks for primary no-AI gain models.**

| **Outcome gain model** | **Full adjusted model fit** | **Baseline-profile adjustment** | **Section adjustment** | **Interpretive summary** |
| --- | --- | --- | --- | --- |
| Harm-sensitive AI literacy, posttest gain | Model R2 = .747 | Early reasoning profile included; climate-control advantage retained | Section included as fixed blocking factor; direction retained in alternative checks | Climate-control remained higher than both comparison conditions. |
| Harm-sensitive AI literacy, delayed gain | Model R2 = .673 | Early reasoning profile included; climate-control advantage retained | Section included as fixed blocking factor; direction retained in alternative checks | Delayed transfer pattern remained consistent with the main interpretation. |
| Argumentative writing quality, posttest gain | Model R2 = .556 | Early reasoning profile included; climate-control advantage retained | Section included as fixed blocking factor; direction retained in alternative checks | Climate-control retained the strongest immediate writing-quality gains. |
| Argumentative writing quality, delayed gain | Model R2 = .540 | Early reasoning profile included; climate-control advantage retained | Section included as fixed blocking factor; direction retained in alternative checks | Climate-control retained the strongest delayed writing-quality gains. |

**Table A2. HARM-CAL computational model documentation.**

| **Component** | **Available documentation** | **Reproducibility status** | **Interpretive use** |
| --- | --- | --- | --- |
| Transformer-family classifier | Unit-level probabilities, thresholded predictions, fold assignments, and performance summaries are available. | The exact pretrained checkpoint and original transformer training script were not retained. | Reported as an archived classifier-output benchmark. |
| TF-IDF logistic-regression baseline | Unigram and bigram TF-IDF features, minimum document frequency of 2, maximum of 8,000 features, balanced class weights, and one-vs-rest logistic regression are documented. | Fully specified as the transparent reproducible baseline. | Used to interpret the added value of the transformer-family classifier. |
| Cross-validation structure | Participant-level five-fold cross-validation splits are available. | Reproducible at the participant-split level. | Prevents units from the same learner from appearing in both train and test folds. |
| Human annotation foundation | HARM-CAL double-coded units and reliability summaries are available. | Reproducible at the annotation-summary level. | Supports interpretation of classifier labels as language-based reasoning-state markers. |

**Table A3. Uptake traceability evidence.**

| **Condition** | **Direct uptake** | **Revised uptake** | **Rejected uptake** | **Independently re-authored uptake** |
| --- | --- | --- | --- | --- |
| Standard ChatGPT | High direct uptake in the main analysis | Common | Present | Low |
| Climate-control ChatGPT | Low direct uptake in the main analysis | Common | Present | High |

The uptake records were analyzed as traceable movements from ChatGPT output into learner drafts. The available file contains one adjudicated category per trace rather than separate coder fields, so uptake is interpreted as behavioral process evidence. This evidentiary status is distinct from the HARM-CAL label set, for which independent double-coding statistics are reported in Table 7.
